# Epstein-Barr virus and telomerase: from cell immortalization to therapy

**DOI:** 10.1186/1750-9378-9-8

**Published:** 2014-02-26

**Authors:** Riccardo Dolcetti, Silvia Giunco, Jessica Dal Col, Andrea Celeghin, Katy Mastorci, Anita De Rossi

**Affiliations:** 1 Cancer Bio-Immunotherapy Unit, CRO Aviano, National Cancer Institute, Aviano, PN, Italy; 2 Viral oncology Unit, Section of Oncology and Immunology, Department of Surgery, Oncology and Gastroenterology, University of Padova, IOV-IRCCS, Padova, Italy

**Keywords:** Epstein-Barr virus, Telomerase, Cancer, Lymphoma, Lytic replication, Antivirals

## Abstract

Overcoming cellular senescence is strictly required for virus-driven tumors, including those associated with Epstein-Barr virus (EBV). This critical step is successfully accomplished by EBV through TERT expression and telomerase activation in infected cells. We herein review the complex interplay between EBV and TERT/telomerase in EBV-driven tumorigenesis. Evidence accumulated so far clearly indicates that elucidation of this issue may offer promising opportunities for the design of innovative treatment modalities for EBV-associated malignancies. Indeed, several therapeutic strategies for telomerase inhibition have been developed and are being investigated in clinical trials. In this respect, our recent finding that TERT inhibition sensitizes EBV+ lymphoma cells to antivirals through activation of EBV lytic replication is particularly promising and provides a rationale for the activation of clinical studies aimed at assessing the effects of combination therapies with TERT inhibitors and antivirals for the treatment of EBV-associated malignancies.

## Introduction

More than 90% of the world’s population is infected by Epstein-Barr virus (EBV), a ubiquitous gammaherpesvirus that may silently persist in memory B lymphocytes. Primary infection is usually asymptomatic and when it is delayed until adolescence or adulthood a benign lymphoproliferative disease, known as infectious mononucleosis, may occur. B lymphocytes are the main target of EBV infection *in vivo*, although epithelial cells, and T or Natural Killer (NK) cells may also carry the virus. Infection of B lymphocytes is usually non-productive or latent, whereas intermittent reactivation and virus replication at epithelial surfaces allow the spreading of EBV to new hosts. Despite its widespread diffusion and apparent harmlessness, EBV is causally linked to the development of both lymphoid and epithelial malignancies, including Burkitt lymphoma (BL), Hodgkin lymphoma, post-transplant lymphoproliferations, AIDS-associated lymphomas, nasopharyngeal and gastric carcinoma [1]. EBV infection of primary B lymphocytes results in permanent growth of these cells, an effect promoted by the full spectrum of EBV-encoded latency proteins, including six EBV nuclear antigens (EBNAs) and three latent membrane proteins (LMP-1, LMP-2A, LMP-2B), the so called latency III program, which can be found in EBV-transformed lymphoblastoid cell lines (LCLs), in post-transplant lymphoproliferations and in AIDS-associated immunoblastic lymphomas [1,2]. A second latency program (type II) can be detected in tumor cells of Hodgkin lymphoma and in nasopharyngeal carcinoma, which express only EBNA-1 and the LMPs, whereas the most restricted form of EBV latency is characteristic of BL lymphoma in which only EBNA-1 is expressed. In all forms of latency, EBV expresses the EBERs, small non-polyadenylated, non-coding double-strand RNAs, which may also contribute to EBV-driven B-cell immortalization [3]. EBV may activate the lytic replication program upon terminal differentiation of EBV-infected memory B lymphocytes into antibody-secreting plasma cells. Although well equipped to promote the growth of B lymphocytes, EBV may drive the proliferation of these cells only transiently in immunocompetent hosts. This is due to the existence of a complex, strictly regulated immunological control involving various humoral and cellular effectors of immunity. Through the long time evolutionary adaptation in humans, the virus has evolved several potent mechanisms by which the type III cells that express the growth program evade the immune response. This may also explain why only a limited proportion of EBV-seropositive individuals develop EBV-associated lymphomas, even in the setting of immune deficiency.

Studies carried out with recombinant EBV strains lacking individual latent genes allowed the identification of the genes that are essential for B-cell immortalization *in vitro*. Available evidence indicates that EBNA-2 and LMP-1 are absolutely required for EBV-mediated B-cell transformation, whereas a crucial role is played by EBNA-1, EBNA-3, -5, and -6 [1]. Full immortalization is achieved through the concerted action of several EBV proteins that derange cellular pathways controlling growth and/or survival. These viral proteins usually act cooperatively and may induce different biologic effects in different cellular backgrounds. LMP-1 is considered the major EBV oncoprotein, acting as an oncogene in rodent fibroblast cells [4,5]. LMP-1 functions as a constitutively active tumor necrosis factor receptor, mimicking an activated CD40 receptor, although structurally different [6,7]. LMP-1 has pleiotropic functions being able to promote B-cell activation, homotypic and heterotypic cell adhesion and the expression of cell surface (i.e. CD23, CD39, CD40 and CD44) and adhesion (LFA1, ICAM1 and LFA3) molecules. LMP-1 is also responsible for the up-regulation of anti-apoptotic proteins, and may suppress cellular senescence. Particularly relevant from an oncogenic point of view is the ability of LMP-1 to activate multiple cellular signaling pathways, including mitogen-activated protein kinase (MAPK), c-Jun N-terminal kinase (JNK), phosphatidylinositol 3-kinase (PI3K)/Akt, and NF-κB [8].

LCLs obtained by EBV infection *in vitro* of B lymphocytes, is considered a useful model to investigate the relationships between virus and host in the EBV-driven lymphomagenesis. Indeed, these immortalized cells are similar to those regularly produced *in vivo* but negatively controlled by EBV-specific CD8+ cytotoxic T cells [9]. Studies using this model assess that EBV-driven malignancies and LCLs selectively express latent viral proteins and maintain their ability to grow indefinitely through inappropriate activation of TERT. Expression of latent EBV proteins is not sufficient to fully immortalize EBV-infected B cells. Only EBV-carrying B cells with sustained telomerase activity are truly immortalized, whereas telomerase-negative cells, although exhibiting a prolonged life-span, eventually undergo cellular senescence and terminate their life span through the shortening of their telomeres [10,11]. The finding that the majority of EBV-driven tumors *in vivo* are telomerase-positive confirms the relevance of telomerase expression in the process of tumorigenesis.

### Pathogenic role of telomerase in cell immortalization and transformation

Genetic instability is a hallmark of cancer and tumor cells should circumvent replicative senescence and acquire the ability to sustain unlimited proliferation. Telomere/telomerase interplay is an important mechanism involved in the genomic stability and cellular replicative potential and its dysfunction has emerged as playing a key role in carcinogenesis [12]. Telomeres are specialized DNA structures located at the end of chromosomes and are essential in stabilizing chromosomes by protecting them from end-to-end fusion and DNA degradation. Telomeres are composed of (TTAGGG)n tandem repeats associated with the telomere-binding proteins TRF1, TRF2, RAP1, TIN2, TPP1 and POT1, which constitute the shelterin complex [13]. Telomeres are progressively shortened during each cell division by replication-dependent loss of sequences at DNA termini due to the failure of DNA polymerase to completely replicate the 3′ end of chromosomes [14]. When telomeres become critically short (the Hayflick limit), cells undergo replicative senescence and apoptosis; further erosion of telomeres may impair their function in protecting chromosome ends, resulting in genetic instability. Nonetheless, cell division-associated telomere shortening prevents unlimited cell proliferation and, thus, tumour development/progression. To escape this proliferation barrier, cells must stabilize their telomeres. Most tumors maintain their ability to grow indefinitely through inappropriate expression of telomerase, a ribonucleoprotein complex containing an internal RNA template (TR), used as a template for elongation of telomeres, and the protein with telomere-specific reverse transcriptase activity (telomerase reverse transcriptase [TERT]) [15]. While TR has broad tissue distribution and is constitutively present in normal and tumour cells, TERT is the rate-limiting component of the telomerase complex, and its expression generally well correlates with telomerase activity. Over-expression TR along with TERT may increase telomerase activity, while specific TR variants may reduce its association with TERT, thus diminishing the telomerase activity in telomere lengthening [16]. Expression of TERT is generally restricted to stem cells, and is usually repressed in normal somatic cells. It may be expressed at low levels in normal hematopoietic cells according to their state of differentiation/activation. In contrast, TERT is expressed in the vast majority of immortalized and fully transformed cells. TERT is essential for unlimited cell growth, and thus plays a critical role in tumor formation and progression (*reviewed in*[17]).

Regulation of telomerase operates at several biological levels: transcription, mRNA splicing, sub-cellular localization of each component, and assembly of TR and TERT in an active ribonucleoprotein complex. Transcription of *TERT* gene is likely the key determinant in the regulation of telomerase activity; TERT transcriptional activity is specifically up-regulated in cancer cells, but it is silent in most normal cells. More than 20 transcription factor-binding sites acting as activators or repressors have been identified within the TERT promoter. Cooperation of MYC and SP1 is required for full activation of the TERT promoter, while TP53, through its interaction with SP1, down-regulates TERT. *TERT* is also directly activated by nuclear factor (NF)-kB, hypoxia-inducible factor-1, and the ETS/MYC complex. The histone methyltransferase SMYD3 also directly contributes to inducible and constitutive TERT expression in normal and malignant human cells. TERT expression is suppressed by the oncosuppressor genes *WT127* and *MEN1,* and through the MAD/MYC and the TGF-β/SMAD pathways. Cell cycle inhibitors p16INK4a and p27KIP1 have also been shown to down-regulate TERT expression in cancer cells (*reviewed in*[17]). Regulation of *TERT* transcription may also involve DNA methylation, as the *TERT* promoter contains a cluster of CpG sites. At post-transcriptional level, modulation of telomerase may occur by alternative splicings; at least 10 different variants of TERT mRNA have been described, and some of these splicing products have been proposed to exert a dominant negative function by competitive interaction with components of the telomerase complex [18]. Telomerase activity is also controlled through post-translational modifications of the TERT protein. Phosphorylation of the protein at critical sites by the PI3K/AKT kinase pathway seems to be crucial for telomerase activity. Telomere-binding proteins, Telomeric Repeat binding Factor (TRF)1, TRF2, Repressor/Activator Protein1 (RAP1), TRF1-interacting Nuclear protein 2 (TIN2), TTP1 (also known as TINT1, PTOP, PIP1), and Protection Of Telomers 1 (POT1), which constitute the shelterin complex, play a role in the activity of telomerase; TPP1 heteromerizes with POT1; the POT1-TPP1 complex is capable of recruiting and stimulating telomerase activity, thereby regulating telomere length through TPP1-telomerase interaction [19]. Notably, recent studies have suggested that, besides maintenance of telomere length, TERT is involved in several other cell functions. Expression of TERT increases replicative kinetics [20,21], promotes cell growth in adverse conditions and may also act as an anti-apoptotic agent [22-24]. High levels of telomerase confer resistance to several antineoplastic drugs [25,26].

### Interplay between EBV infection and telomerase activation in EBV-driven tumors

Although it is well recognized that the establishment of latent EBV infection and TERT activation are both required for EBV-driven cell transformation, temporal and possible causal relationships between these two events remain to be clarified. Early passages EBV-infected B lymphocytes greatly differed in their timing of TERT expression and telomerase activation; EBV-driven B-cell activation may fail in the induction of telomerase activity and telomerase-negative EBV-infected B cells may have a prolonged life-span compared to normal B lymphocytes [10,20]. Although EBV-infected B cells exhibit higher proliferative activity than resting primary B lymphocytes, very few EBV-carrying B cells will eventually progress to immortalization, most of them reaching proliferative crisis and ending their lifespan even after 150 population doubling levels depending on genetic factors, including telomere length. Only LCLs developing a strong telomerase activity associated with aneuploidy overcome cellular crisis and become stably immortalized. Therefore additional changes at the cellular level are required to cooperate with the latent EBV proteins during immortalization of EBV-infected B cells [10]. It has been recently suggested that telomere length may be maintained in EBV-infected B lymphocytes by an alternative lengthening of telomeres (ALT) [27]. However, lack of TERT activation precluded their long-term establishment in culture and their immortalization [20].

In primary B lymphocytes, activation of TERT occurs concomitantly with the induction of latent EBV proteins and down-regulation of EBV lytic gene expression [20]. We have demonstrated that LMP-1 activates TERT at the transcriptional level *via* NF-kB and MAPK/ERK1/2 pathways [28]. LMP-1 induces telomerase activity also in nasopharyngeal carcinoma cells, an epithelial tumor closely associated with EBV infection. In these cells, LMP-1 up-regulates telomerase expression and phoshorylation through the AKT pathway [29]. In epithelial cells, TERT expression may be also MYC-dependent since mutagenesis of MYC-responsive E-box elements in the TERT promoter inhibited TERT transactivation induced by LMP-1 [30]. In B cells, however, MYC is not involved in mediating the hTERT expression and telomerase activation induced by LMP-1, since MYC silencing does not inhibit LMP-1-induced telomerase activation, and mutagenesis in the NF-κB binding sites, but not in the MYC binding sites, inhibits LMP-1-induced activation of the TERT promoter [28]. This is of particular interest considering that, while in EBV-negative BL the translocated and over-expressed MYC plays a key role in TERT activation, in EBV-positive diffuse large B-cell Lymphomas and immunoblastic lymphomas where *MYC* is in germ-line configuration, TERT is likely to be activated by LMP-1, as it occurs in LCLs [28].

Notably, in addition to maintaining telomere length and allowing EBV-infected cells to overcome senescence and apoptosis, TERT may promote EBV-driven lymphomagenesis through extra-telomeric functions. One interesting result achieved *in vitro* using the LCL model is that TERT expression plays a relevant role in inhibiting the virus lytic cycle, thereby favouring the induction and maintenance of EBV latency in primary B lymphocytes, a prerequisite for EBV-driven transformation. Indeed, high level of endogenous TERT expression or ectopic TERT expression on telomerase-negative EBV-infected cells prevents the induction of viral lytic cycle. By contrast, TERT silencing by specific siRNA or short-hairpin (sh)RNA induced the expression of BZLF1, EA-D, and gp350 EBV lytic proteins and triggered a complete lytic cycle. This occurs in both EBV-immortalized LCL and fully transformed EBV-positive BL cell lines thus supporting the notion that TERT is a critical regulator of the balance between viral latent and lytic cycles [20,31]. Moreover, TERT inhibition induced an accumulation of cells in the S phase, an effect likely due to the dephosphorylation of 4EBP1, an AKT1-dependent substrate, which results in a decreased availability of proteins needed for cell cycle progression. Besides inducing cell death through activation of complete EBV lytic replication, TERT inhibition triggered AKT1/FOXO3/NOXA-dependent apoptosis in EBV-positive and EBV-negative BL cell lines [31].

**Figure 1:**
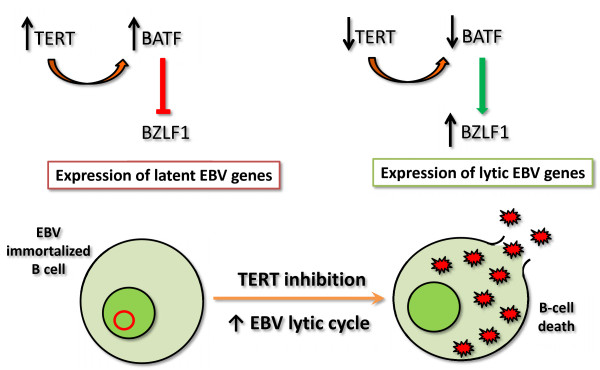
**TERT inhibition induces EBV lytic cycle.** In EBV-infected B cells, TERT up-regulates the expression of BATF, a negative regulator of BZLF1, thus preserving EBV latency. Following TERT inhibition, BATF level significantly decreases; the expression of BZLF1 leads to the induction of lytic proteins and a complete EBV lytic cycle.

The fine mechanism(s) by which TERT prevents the expression of lytic proteins is still an interesting open question. It has been demonstrated that the treatment of primary EBV-positive BL with zidovudine (AZT), a thymidine analog, induced EBV lytic cycle and cell death through the NF-κB pathway [32,33]. Given that AZT may inhibit telomerase activity [34], this finding further supports the strong relationship between TERT level and EBV latent/lytic status and it may suggest that this occurs via NF-κB pathway. To shed light on the possible mechanisms underlying the activation of EBV lytic replication induced by TERT inhibition, a study investigated the involvement of BATF, a transcription factor, which negatively regulates AP-1 activity [35,36]. BATF has been shown to inhibit the expression of BZLF1, thus reducing EBV lytic replication in latently infected B cells [37]. It has been shown that ectopic expression of TERT in B cells significantly increased BATF expression, whereas TERT silencing by shRNA decreased BATF mRNA levels and protein expression and induced the expression of lytic protein [31]. These results suggest that TERT silencing promotes the activation of EBV replication by reducing inhibition of BATF-driven BZLF1 transcription (Figure 1). As viral lytic replication is associated with the death of infected cells, TERT inhibition may be promising strategy to treat EBV-driven malignancies.

### Telomerase as a promising therapeutic target for human tumors

The high expression of TERT in tumor cells and the requirement of a sustained telomerase activity for their unlimited proliferation capability make telomerase a particularly attractive target for cancer therapy. Moreover, the fact that rapidly proliferating cancer cells have shorter telomeres compared to normal somatic cells and stem cells further corroborates the enhanced specificity for tumor cells of the cytotoxic effects exerted by drugs targeting telomerase [38]. Several strategies targeting telomerase are being explored at the pre-clinical level, and two major approaches are currently under clinical investigation. Telomerase can be directly targeted by drugs able to inhibit TERT activity or its RNA template. Alternatively, drugs such as G-quadruplex stabilizers, tankyrases or HSP90 inhibitors may act indirectly on telomerase by preventing its access to telomeres or inhibiting binding of telomerase-associated proteins leading to telomere uncapping and cell apoptosis [39]. GRN163L (Imetelstat) is a highly promising drug able to inhibit telomerase activity by acting as a direct telomerase RNA template antagonist rather than behaving as a normal antisense oligonucleotide [40]. This compound has been successfully tested in several phase I studies involving patients with non-small cell lung cancer, locally recurrent or metastatic breast cancer, multiple myeloma or essential thrombocythemia and its efficacy is now being investigated in phase II clinical trials [39]. Among small molecule inhibitors targeting hTERT, the non-nucleosidic compound BIBR1532 is particularly interesting due to its ability to inhibit telomerase by non-competitively binding to the active site of TERT [41]. Preclinical studies carried out with cell lines of different tumor histotypes demonstrated that BIBR1532 can inhibit telomerase activity and induce cell growth arrest without causing acute cytotoxicity. Evidence has been also provided indicating that BIBR1532 can sensitize tumor cells to chemotherapy [42,43]. T-oligo is a newly developed drug composed of a single stranded 11-bp oligonucleotide with sequence homology to telomeres able to potently induce DNA damage responses including apoptosis, differentiation and senescence [44,45]. Notably, in normal cells, T-oligo induces only transient antiproliferative effects due to the presence of functional cell cycle check points [45]. Given that telomere length depends on the balance between progressive loss during cell proliferation and extension induced by telomerase activation, a deeper knowledge of telomere/telomerase interplay is critical to choose the most appropriate timing for administering telomerase-targeted drugs. This is a highly relevant issue to design the most effective schedules of treatment including these innovative drugs.

A second main therapeutic approach involves immunotherapy strategies targeting telomerase [39]. Considering that most cancers express telomerase, peptides derived by this protein can be considered as universal tumor-associated antigens. Indeed, evidence accumulated so far clearly indicates that telomerase-derived peptides may elicit specific CD8+ and CD4+ T cell responses of potential clinical relevance [46,47]. Several strategies are being investigated to enhance the immunogenicity of TERT-based vaccines, including the use of adjuvants like GM-CSF or TLR-7 (GV1001 vaccine) [48] or the generation of cryptic peptide vaccines, such as Vx-001 [49]. In these latter vaccines, one amino acid residue of a TERT peptide is replaced for another resulting in a higher affinity to the presenting HLA molecule and a more efficient stimulation of TERT-specific T cells [49]. In addition to these peptide-based vaccines, immunotherapy approaches targeting TERT also include dendritic cell-based vaccines, which do not suffer from the limitations imposed by HLA restriction. GRNVAC1 is a vaccine composed of immature dendritic cells transfected *ex vivo* with a chimeric mRNA encoding the entire TERT sequence and a portion of the lysosomal-associated membrane protein, which redirects the TERT protein to the lysosome degradation pathway favoring its degradation into immunogenic peptides [50]. Several TERT-targeting vaccines have successfully completed phase I/II studies and are now being investigated in phase III clinical trials.

### Strategies exploiting telomerase/TERT inhibition for the treatment of EBV-driven tumors

The observation that EBV-driven tumors generally express telomerase at high levels provides the rationale supporting the use of the aforementioned pharmacological and immunological approaches targeting telomerase/TERT also for the treatment of these malignancies. Nevertheless, exploitation of telomerase inhibition in this setting may offer additional advantages of potential clinical relevance. In fact, our observation that inhibition of TERT by shRNA triggers virus replication in both EBV-immortalized and fully transformed B cells [31] provides the highly promising opportunity to combine TERT inhibition with antiviral drugs to improve the rate of clinical responses. Indeed, there is an increasing interest in developing strategies able to reactivate EBV lytic gene expression in latently infected tumor cells for the treatment of overt EBV-associated lymphomas. In fact, lytic infection may promote the death of EBV-positive lymphoma cells in vivo, an effect that may be therapeutically relevant since it also favours immune recognition of viral antigens that further enhances the killing of tumor cells. Therefore, drugs targeting cells undergoing viral replication may be used not only to prevent or contain the spreading of EBV infection, but also mainly for their cytotoxic activity on the infected cells and the adjacent cells [51]. Several chemotherapeutic drugs are known to trigger EBV replication, and combination of antivirals with lytic cycle inducers is emerging as a promising strategy for the treatment of EBV-driven lymphomas [52]. We have recently demonstrated that ganciclovir markedly enhances the the anti-proliferative and pro-apoptotic effects induced by TERT inhibition in both EBV-positive BL cells and LCLs [31]. This is probably related to induction of EBV replication and may be the result of the activation of the pro-drug by EBV lytic products such as the viral thymidine kinase or viral protein kinase. In this respect, drugs able to inhibit TERT may be regarded as sensitizers for the activity of antivirals. On these grounds, the combination of antiviral drugs with strategies able to inhibit TERT expression/activity may result in therapeutically relevant effects in patients with EBV-related malignancies. This possibility appears particularly promising in light of the recent development of potent and specific telomerase inhibitors.

In EBV-associated tumors, telomerase can be also targeted indirectly by strategies that inhibit the expression of LMP-1, the major viral oncoprotein endowed with pleiotropic effects also including the ability to up-regulate TERT at the transcriptional level [28,29]. In this respect, it has been demonstrated that inhibition of LMP-1 expression by a DNAzyme (Dz1) able to cleave LMP-1 mRNA down-regulates the expression of the catalytic subunit of telomerase (TERT), both at the protein and mRNA levels, and consequently inhibits telomerase activity in LMP1-positive nasopharyngeal carcinoma cells [29]. Similar strategies may be adopted with therapeutic purposes also for EBV-driven lymphomas.

## Conclusions

Available evidence indicates that TERT expression and telomerase activation play a critical role in EBV-driven tumorigenesis. Several therapeutic strategies for telomerase inhibition have been developed and are being investigated in clinical trials, although data concerning the response rates obtained in patients with EBV-associated lymphomas are limited. Most therapeutics have shown to be more effective when used in combination with standard therapies. In this respect, our finding that TERT inhibition sensitizes EBV+ lymphoma cells to antivirals through activation of EBV lytic replication is particularly promising and provides a rationale for conducting further research to assess the effects of combination therapies with TERT inhibitors and antivirals for the treatment of EBV-associated malignancies.

## Abbreviations

AZT: Zidovudine; ATL: Alternative lengthening of telomerases; BL: Burkitt lymphoma; EBV: EBV nuclear antigens; EBNAs: Epstein-Barr virus; LCL: Lymphoblastoid cell lines; MAPK: Mitogen-activated protein kinase; NK: Natural killer; NF: Nuclear factor; JNK: c-Jun N-terminal kinase; PI3K: Phosphatidylinositol 3-kinase; sh: Short-hairpin; TERT: Telomerase reverse transcriptase.

## Competing interests

The authors declare that they have no competing interests.

## Authors’ contributions

All authors performed the literature research, composed the article and approved the final version to be submitted. All authors read and approved the final manuscript.
